# The Evolutionary Genomics of Serpentine Adaptation

**DOI:** 10.3389/fpls.2020.574616

**Published:** 2020-12-16

**Authors:** Veronika Konečná, Levi Yant, Filip Kolář

**Affiliations:** ^1^Department of Botany, Faculty of Science, Charles University, Prague, Czechia; ^2^Institute of Botany, The Czech Academy of Sciences, Pru˚honice, Czechia; ^3^Future Food Beacon and School of Life Sciences, University of Nottingham, Nottingham, United Kingdom; ^4^Natural History Museum, University of Oslo, Oslo, Norway

**Keywords:** serpentine, adaptation, population genomics, edaphic extremes, ionomics

## Abstract

Serpentine barrens are among the most challenging settings for plant life. Representing a perfect storm of hazards, serpentines consist of broadly skewed elemental profiles, including abundant toxic metals and low nutrient contents on drought-prone, patchily distributed substrates. Accordingly, plants that can tolerate the challenges of serpentine have fascinated biologists for decades, yielding important insights into adaptation to novel ecologies through physiological change. Here we highlight recent progress from studies which demonstrate the power of serpentine as a model for the genomics of adaptation. Given the moderate – but still tractable – complexity presented by the mix of hazards on serpentine, these venues are well-suited for the experimental inquiry of adaptation both in natural and manipulated conditions. Moreover, the island-like distribution of serpentines across landscapes provides abundant natural replicates, offering power to evolutionary genomic inference. Exciting recent insights into the genomic basis of serpentine adaptation point to a partly shared basis that involves sampling from common allele pools available from retained ancestral polymorphism or *via* gene flow. However, a lack of integrated studies deconstructing complex adaptations and linking candidate alleles with fitness consequences leaves room for much deeper exploration. Thus, we still seek the crucial direct link between the phenotypic effect of candidate alleles and their measured adaptive value – a prize that is exceedingly rare to achieve in any study of adaptation. We expect that closing this gap is not far off using the promising model systems described here.

## Introduction

Local adaptation optimizes fitness to the environment, often at the scale of meters. The resultant spatially varying selection leads to between-population genomic divergence that, depending on the intensity of gene flow, may maintain intraspecific adaptive diversity or lead to ecological speciation (Rundle and Nosil, 2005; Savolainen et al., 2013). In sessile plants, heterogeneous landscape mosaics, such as mountains or patchy soils, can trigger dramatic cases of local adaptation, especially in the presence of a steep gradient in the selective agent (Jain and Bradshaw, 1966). However, despite recent progress in the genomics of adaptation, there are still a few empirical inquiries into spatially varying selection (e.g., Hämälä et al., 2018; Hämälä and Savolainen, 2019) that provide empirical verification of the theory concerning adaptation under migration scenarios *via* finding correlation between fitness and environmental factors underlying local selection in natural populations (Yeaman and Whitlock, 2011). Only few studies assess the fine-scale genomic architecture of complex adaptive traits adequately (Holliday et al., 2016).

**Figure 1:**
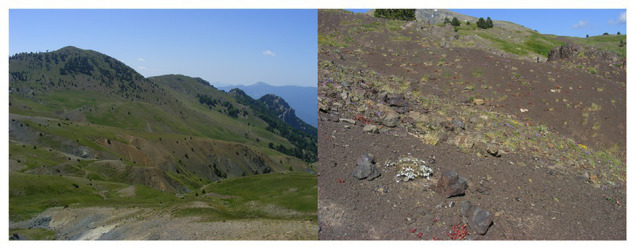
Extreme environment of serpentine barrens, illustrating sharp boundaries to adjacent land and low productivity (Pindos Mountains, Greece). Photos: F. Kolář.

Serpentine barrens (Figure 1) represent powerful models to understand genome modification to local conditions because their extreme chemical and physical properties act as strong, quantifiable selective pressures. Derived from ultramafic rocks, serpentine soils are highly skewed in their content of many elements, being typically: (i) low in macronutrients such as Ca, K, N, and P, (ii) high in metals Co, Cr, and Ni, and (iii) greatly reduced in Ca relative to Mg. Worldwide, it is this highly skewed Ca/Mg ratio that defines serpentines (O’Dell and Rajakaruna, 2011), despite considerable diversity in other qualities. To add insult to injury, serpentine soils are typically very porous, with low water holding capacity and, due to their dark color, are frequently prone to substrate over-heating (Proctor and Woodell, 1975; Brady et al., 2005). These multifarious chemical and physical characteristics together have been termed “the serpentine syndrome” (Jenny, 1980), a state that results in very low ecosystem productivity with low competition and frequent endemism (Kruckeberg, 1954; Whittaker, 1954; Brady et al., 2005; Harrison and Rajakaruna, 2011). Moreover, the island-like distribution of serpentines provides abrupt edaphic contrasts that are replicated frequently across landscapes, triggering parallel adaptation (Roberts and Proctor, 1992). Such natural replicates can be leveraged to discern consistent trends in mechanisms and genetic bases of adaptation, as well as ecological speciation (Rundle and Nosil, 2005; Losos, 2011).

The distinctive floristic composition of serpentines attracted botanists as early as the beginning of the twentieth century. What started as a general fascination with floristic peculiarities (Pančić, 1859; Lämmermayr, 1927; Novák, 1928; reviewed by Whittaker, 1954; Eggler, 1955) continued with experiments testing local adaptation (Kruckeberg, 1951, 1954) and targeted genetic investigations (Bradshaw, 2005; Bratteler et al., 2006). Contributions have emerged from a broad diversity of species, including *Achillea, Cerastium, Collinsia, Gilia, Helianthus, Knautia, Mimulus, Silene, and Streptanthus* (reviewed by Brady et al., 2005; O’Dell and Rajakaruna, 2011; Rajakaruna, 2018), significantly contributing to our understanding of the importance of local adaptation and ecotypic differentiation in plants.

As historical context is well summarized elsewhere (e.g., Brady et al., 2005; Anacker, 2014), we here focus on recent advances providing context for genomic studies. We first highlight advances in our understanding of serpentine adaptation at the phenotypic, physiological, and genomic levels. We discuss: (i) advances in knowledge of the selective factors imposed by serpentines, (ii) progress in experimental verification of local adaptation to serpentine soils, and (iii) plant responses to serpentines: the “phenotype” of serpentine adaptation. Finally, (iv) we summarize the first genomic studies that have very recently been built on previous insights and outline ways forward to integrate the study of serpentine adaptation.

## Drivers of Selection at Serpentine Soils

Given the heterogeneity between various serpentine sites, in order to understand the mechanistic basis of serpentine adaptation it is first necessary to define the exact selective agents in play for any given case. While serpentine syndrome represents a complex set of selection pressures that vary from site to site, since the 1950’s there has been broad evidence that Ca availability plays a leading role (Vlamis and Jenny, 1948; Vlamis, 1949; Kruckeberg, 1954; Walker et al., 1955). More recently, the advent of high-throughput inductively coupled plasma mass spectrometry (ICP-MS; “ionomics” – Salt et al., 2008; Huang and Salt, 2016) has accelerated the characterization of that ‘hidden half’ of the plant environment: the underground soil matrix. Ionomics has allowed the rapid characterization of elemental accumulation in plant tissues in common garden experiments in diverse soil types from serpentines, to toxic mines, to saline soils (e.g., Arnold et al., 2016; Stein et al., 2017; Busoms et al., 2018; Preite et al., 2019). This and other recent advances in soil profiling have supported the salient role of distorted Ca/Mg ratio on serpentines (Palm and Van Volkenburgh, 2014), but has also identified other players, such as elevated heavy metal concentrations (Co, Cr, Ni, and/or Zn), for example in *Arabidopsis lyrata* (Veatch-Blohm et al., 2017) or *Knautia serpentinicola* (Čertner et al., 2018). These elevated metal levels are sometimes accompanied by lower concentrations of nutrients K, P, and S, as seen in *Helianthus exilis* (Sambatti and Rice, 2006), *Cerastium alpinum* (Berglund et al., 2004), and *Arabidopsis arenosa* (Arnold et al., 2016). Additional factors, such as drought, can interact with major chemical factors contributing to local adaptation to specific stresses, especially in drought-prone areas (e.g., Salehi Eskandari et al., 2017). The roles of biotic interactions, such as with bacteria, archaea, or mycorrhiza, are largely unknown with the little available evidence suggesting highly diverse effects (Mengoni et al., 2001; Pal and Paul, 2004; Davoodian et al., 2012; Doubková et al., 2012; reviewed by Schechter and Branco, 2014 and Southworth et al., 2014). For instance, higher diversity of arbuscular mycorrhizal fungi (AMF) was observed in serpentine populations of *Collinsia sparsiflora* compared to non-serpentine ones (Schechter and Bruns, 2008). Further, AMF more efficiently promoted growth and P uptake in serpentine *K. serpentinicola* (Doubková et al., 2012). On the other hand, the bacterial communities from serpentine and non-serpentine soils in Northern California were not different from each other (Oline, 2006).

To add to this complexity, there commonly exists fine-scale, site-specific differences in the serpentine-defining factors themselves. Indeed, Berglund et al. (2004) leveraged such variability among different serpentine sites occupied by *C. alpinum* to demonstrate that strength of tolerance to Mg and Ni was related particularly to effective concentrations of these elements in soil at each site. Similarly, the relative roles of other components depend on particular species – or even the site – studied. For example, variation in B, Ca, Fe, Na, and Zn is observed between serpentine barrens harboring *Mimulus guttatus* (Selby and Willis, 2018). In line with this, differences in physical properties also seem to be regionally specific rather than a universal property of all serpentines. For example, while drought and erosion characterize serpentines in drought-prone regions such as California or the Middle East (Kruckeberg, 1984; Salehi-Eskandari et al., 2018), they do not distinguish serpentine and non-serpentine sites of otherwise similar geomorphology in Central and Northern Europe (Novák, 1928; Rune, 1953), (Teptina et al., 2018), and tropical regions in South and Southeast Asia (Galey et al., 2017).

In summary, the study of serpentine adaptation requires an initial decision: one must choose whether to address serpentine adaptation holistically (including physical properties, biotic interactions, and site-specific soil chemistry) or to instead focus on a universally dominating parameter (such as altered Ca/Mg ratio or elevated Ni content).

## Experimental Evidence for Adaptation to Serpentine Soils

**Figure 2:**
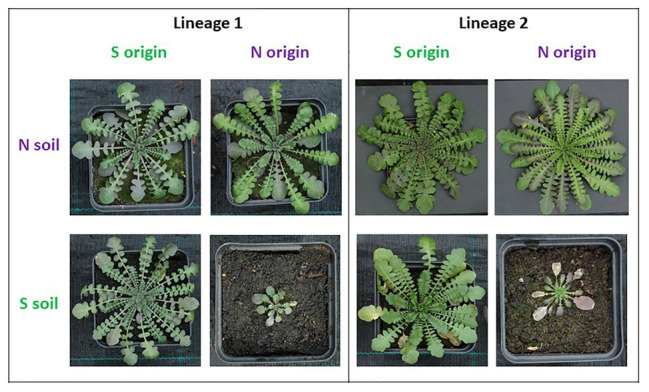
Transplant experiment demonstrating parallel fitness response of two lineages of *Arabidopsis arenosa* to serpentine soil, depending on the substrate of origin. Two pairs of originally serpentine (S) and non-serpentine (N) populations representing different genetic lineages were cultivated in their native and foreign (native to the other member of the pair) soil, illustrative photo of representatives from all populations and treatments are depicted. Note the considerably smaller plants of non-serpentine origin when cultivated in serpentine soil but lack of such response for serpentine population in the non-serpentine soil suggesting the absence of the substrate-related trade-offs for serpentine plants. Photos by V. Konečná.

A long tradition of reciprocal transplant experiments since the 1950’s (e.g., Kruckeberg, 1950, 1951, 1954, 1967) provided broad evidence of local adaptation to serpentine soils. These approaches have recently been expanded to diverse species, e.g., *Helianthus exilis* (Sambatti and Rice, 2006), *Collinsia sparsiflora* (Wright et al., 2006), *Achillea millefolium* (O’Dell and Claassen, 2006), *Mimulus guttatus* (reviewed by Selby et al., 2014; Selby and Willis, 2018), and *Arabidopsis arenosa* (Figure 2). The observed adaptive differences were compromised of a wide range of fitness proxies, from the extent of juvenile mortality in *Mimulus*, to higher biomass production in *Achillea*, and seed production in *Helianthus*.

Taking our understanding of adaptive differences a step further, specific deconstruction of serpentine tolerance to individual elements has been performed in several contexts. Because serpentine chemical stress can be simply modeled, researchers have modulated the cardinal factors: Ca/Mg ratios and Ni levels in hydroponics and custom growth media (Proctor, 1971; Gabbrielli and Pandolfini, 1984; O’Dell and Rajakaruna, 2011). In *Knautia arvensis* and *Cerastium alpinum*, higher tolerance of serpentine populations to elevated Mg and Ni is evidenced by greater root growth (Berglund et al., 2004; Kolář et al., 2014). A specific effect of Ca/Mg ratio on both total biomass and photosynthetic rates was shown in *M. guttatus* (Palm et al., 2012). Furthermore, the effect of specific elements (Cr, Ni, and Ca/Mg ratio) on seed germination has been examined in *Arabidopsis lyrata* (Veatch-Blohm et al., 2013, 2017). There, while Ni caused a slower seedling growth, especially in non-serpentine accessions, there was no differential response to elevated Cr and Mg that could be related to fitness. Therefore, we can conclude that mechanisms reducing the Ni and/or Mg toxicity evolved in serpentine populations of many species; their phenotypes, such as length of the root, however, differ.

The origins of these phenotypes have been probed with genetic investigations of population history, which have documented striking manifold parallel colonizations of sites for a majority of the sufficiently sampled species or species groups (e.g., *Alyssum serpyllifolium*, Mengoni et al., 2003; Sobczyk et al., 2017; *C. alpinum*, Berglund et al., 2004; *Lasthenia californica* complex, Rajakaruna et al., 2003a; *M. guttatus*, Selby and Willis, 2018; *Minuartia verna* complex, Nunvářová Kabátová et al., 2019; *Solidago virgaurea*, Sakaguchi et al., 2017; *Streptanthus glandulosus* complex, Mayer and Soltis, 1994). In fact, single origins of serpentine populations are very rarely documented among species growing both on and off multiple serpentines (e.g., *Picris hieracioides*, Sakaguchi et al., 2018), and remains rather a property of genuine serpentine endemics (e.g., *K. serpentinicola*, Kolář et al., 2012, *Halacsya sendtneri*, Cecchi and Selvi, 2009). Unfortunately, only in a few cases have such genetic investigations been coupled with reciprocal transplants (Sakaguchi et al., 2017, 2019; Selby and Willis, 2018) or hydroponic experiments (Rajakaruna et al., 2003b,c; Berglund et al., 2004), ultimately demonstrating parallel substrate adaptation across multiple serpentine populations. Such rare cases provide particularly valuable naturally replicated model systems for further, finer-scale investigations of serpentine adaptation.

In other cases, serpentine adaptation is a constitutive trait present in all populations of a species regardless of their native soil chemistry. Such constitutive tolerance towards Ni has been reported for instance in *Silene dioica* (Westerbergh, 1994), *Noccaea goesingense* (Reeves and Baker, 1984), and *Noccaea montana* (Peer et al., 2006). Constitutive tolerance to both high Ni and low Ca/Mg ratios, at least as indicated by root growth, was documented for *Galium valdepilosum*, a likely “pre-adapted” species that colonized nearly all scattered serpentine outcrops throughout its overall species range (Kolář et al., 2014). Moreover, even plants that do not occur on serpentines but grow on dry and nutrient-poor habitats, such as granite outcrops, can tolerate extremely low Ca/Mg ratios, such as *Phacelia dubia* (Taylor and Levy, 2002). Overall, such cases of constitutive tolerance are only rarely documented (reviewed by O’Dell and Rajakaruna, 2011), potentially due to a publication bias towards “positive” results (i.e., where clear within-species local adaptation is evident).

## Mechanisms of Serpentine Adaptation

Experimental studies have revealed a range of life-history and physiological mechanisms potentially underlying serpentine adaptation (Brady et al., 2005; Harrison and Rajakaruna, 2011; O’Dell and Rajakaruna, 2011). Drought stress adaptations include slower growth rate, reduced height, higher root/shoot biomass ratios, early flowering and specific modification of flowers (O’Dell and Rajakaruna, 2011; von Wettberg et al., 2014), and reduced leaf size and sclerophylly observed in some serpentine plants (Brady et al., 2005). In terms of chemistry, selective uptake of some micronutrients and macronutrients such as Ca and exclusion (or regulated accumulation and storage) of different metals are major adaptive mechanisms (Brady et al., 2005; Kazakou et al., 2008). Modulating the status of particular nutrients linked with monitoring uptake thus allows for valuable insight. For example, Kolář et al. (2014) compared serpentine populations of *Knautia serpentinicola* with non-serpentine populations of closely related *Knautia arvensis* in hydroponics with different concentrations of Ni and Mg. Interestingly, serpentine-origin plants accumulated less Ni when cultivated in a high Mg solution. On the contrary, non-serpentine plants accumulated approximately the same concentration of Ni regardless of the Mg concentration in media. Concordantly, in *A. lyrata* under Ni treatment, serpentine-origin plants had lower shoot and root Ni levels compared to non-serpentine plants (Veatch-Blohm et al., 2017).

Given that serpentine is a multi-hazard environment, it is only natural that serpentine adaptation results in physiological changes touching on a range of chemical challenges. A high-throughput approach to assessing this mixture is represented by performing common garden experiments incorporating a broad natural variation in a given species and to measure the relative accumulation of a panel of mineral nutrients. In this way, Arnold et al. (2016) assayed 20 elements in plants from 29 *A. arenosa* populations, after growing them in common conditions. This matrix was contrasted with data from source soil samples, providing a direct comparison of specific natural genetic variation across the ionome. Plants of serpentine origin accumulated the highest levels of K and S, excluded Ni, and exhibited the highest Ca/Mg ratios. These changes indicate a suite of specific, refined physiological adaptations in serpentine *A. arenosa*, which has a genetic basis. In this way, ionomics brings a key tool for understanding the actual “adaptive phenotype” distinguishing serpentine-adapted plants. Such approach, combined with common garden or transplant experiments, should become a standard practice in the characterization of the genetic basis of edaphic adaptation.

Adding to the complexity of multi-hazard adaptation, the rich experimental literature documents multiple solutions to identical environmental triggers (reviewed by Palm and Van Volkenburgh, 2014). For example, plants react to a skewed Ca/Mg ratio with a stunning diversity of mechanisms: either by selective translocation of Ca from roots to shoots (e.g., *A. millefolium*, O’Dell and Claassen, 2006), restriction of Mg uptake (e.g., *Gilia capitata*, Kruckeberg, 1951), or tolerance to higher concentrations of Mg in shoots (e.g., *Streptanthus polygaloides*, Boyd et al., 2009). Analogously, serpentine-adapted plants respond to elevated levels of heavy metals such as Ni in soil either by restricted uptake to shoots (Doubková et al., 2012; Kolář et al., 2014; Salehi Eskandari et al., 2017) or tolerance to high Ni levels in tissues (particularly in metal hyperaccumulators, e.g., Assunção et al., 2003; Galardi et al., 2007; Leigh Broadhurst et al., 2009).

While we have learned much about different adaptation mechanisms at a species level, considerably less is known about the variation in these traits at a population level within species. Experiments leveraging multiple cases of repeated within-species colonization of serpentine patches have demonstrated a broad variation in the strength of responses in a handful of studies (e.g., Berglund et al., 2004; Galardi et al., 2007; Veatch-Blohm et al., 2017). Together with variation in the soil chemistry across serpentine barrens, this suggests that there may be independent solutions even within a species, each fine-tuned to particular conditions at each site.

In summary, because serpentines produce clear challenges that can be experimentally dissected into specific factors (and moreover, which are replicated within species), decades of research have prepared a solid foundation for studies of their genomic basis.

## First Insights into the Genomic Basis of Serpentine Adaptation

Given the multi-challenge nature of serpentines and the diverse adaptive phenotypes generated in response, we may expect a highly complex, polygenic basis. Further, the variation in elemental soil composition between serpentine barrens suggests no single “basis” of serpentine adaptation. On the other hand, as compared to other multi-hazard “extreme” environments (coastal, alpine, or high-arctic sites), serpentines are fairly well-defined, dominated by the effect of few major elements. This, together with strong selective pressures, makes discovery of major-effect candidates seem likely, similar to what has been found for other soils such as metal contaminated and saline sites (e.g., in *Arabidopsis halleri*, Courbot et al., 2007; Willems et al., 2007; in *Noccaea caerulescens*, Deniau et al., 2006; and in *Mimulus guttatus*, Wright et al., 2013). In addition, theory suggests that specific aspects of serpentine colonization – such as abrupt fitness differences and patchily-distributed habitats – favor the emergence of large-effect alleles (Dittmar et al., 2016; Gilbert and Whitlock, 2017). Theory also suggests that adaptation in the face of gene flow, such as that from nearby non-serpentine sites, may promote fixation of smaller numbers of large-effect loci (Yeaman and Whitlock, 2011).

In line with this, the few quantitative trait locus (QTL) studies applied to serpentine ecotypes so far have provided evidence for a simple genetic architecture of single traits (e.g., Ni tolerance in *Silene vulgaris*, Bratteler et al., 2006 and *Caulanthus amplexicaulis*, Burrell et al., 2012). Additionally, studies focused on particular genes known to have an ion homeostasis effect show strong natural differentiation in its sequence variation and/or in associated phenotypic responses (e.g., *A. serpyllifolium*, Sobczyk et al., 2017; *Arabidopsis thaliana*, Bradshaw, 2005; Agrawal et al., 2012). While such hypothesis-driven studies bring valuable insights into the basis of a particular gene or trait, they capture neither genetic architecture nor the complexity of the full serpentine syndrome. Accordingly, neither classical QTL studies nor candidate gene-targeted inquiries can alone provide a picture of the broad genomic remodeling which we speculate is required for robust establishment in such a multi-hazard environment.

In contrast to QTL studies, high-density genomic divergence scans detect signatures of directional selection in a purely natural system from a holistic perspective, both in terms of genes (the entire genome) and parameters screened (the entire serpentine syndrome in nature: both known and unknown factors). In other words, genome scans can be both genetically and phenotypically agnostic. Such scans have been performed in two wild, outcrossing *Arabidopsis* species – *A. arenosa* (Arnold et al., 2016) and *A. lyrata* (Turner et al., 2008, 2010). Both indicate a highly polygenic basis of serpentine adaptation. The study by Turner et al. (2010), notably one of the first truly genome-scale scans for selection used sequencing of pooled samples to reveal outlier differentiated SNPs at loci involved in ion transmembrane transport, metal tolerance, and calcium ion binding. Clearer detection of candidate loci was achieved by individual-level genome resequencing by Arnold et al. (2016), discriminating approximately 160 genes exhibiting multiple signatures of selection in a serpentine-adapted population of *A. arenosa*. These included genes related to Ca signaling, ion homeostasis, metal transport, root macronutrient transport, and dehydration tolerance. These works provided a broad view on the genetic basis of serpentine adaptation and have served as hypothesis generators that can now guide functional assessment of particular alleles in natural conditions.

A complementary study in *M. guttatus* innovatively combined field and genomic approaches by assessing survival differences of F2 mapping populations grown on serpentines by bulk segregant analysis (Selby and Willis, 2018). This enabled the identification of a major QTL (containing several 100 candidate genes) that contributed to the survival of plants by 33%. However, because of the limited size of the survivor pool, the study was underpowered to detect smaller effect loci. Indeed, mapping approaches commonly fail to identify small effect loci and tend to overestimate the influence of large effect loci due to the linkage (Rockman, 2012). Nevertheless, this study was an important step towards linking genomic variation and fitness consequences and demonstrates that the *Mimulus* system has a strong potential to reveal more refined results in the future (Selby et al., 2014).

## Genomics of Ecological Speciation on Serpentine

Ecological speciation can occur when strong divergent selection is associated with the rise of reproductive incompatibilities between ecologically distinct populations (Nosil et al., 2009). Such reproductive incompatibilities may emerge in association with serpentine either as a direct consequence of selection against maladaptive gene flow (e.g., reinforcement to avoid hybrids that are unfit in either environment) or as a by-product of local adaptation, e.g., through physical linkage (as observed in copper-tolerant *Mimulus*, Rajakaruna and Whitton, 2004; Wright et al., 2013) or shifts in flowering time (found in serpentine *Solidago*, Sakaguchi et al., 2018, 2019). Indeed, serpentine barrens provide intriguing candidate cases of incipient – and even parallel – ecological speciation (e.g., Rajakaruna and Whitton, 2004; Moyle et al., 2012; reviewed by Ostevik et al., 2012). The genomic basis of these incompatibilities remains unknown. Aeschbacher et al. (2017) used coalescent modeling on pooled sequence data to detect genome-wide signals of selection against maladaptive gene flow from non-serpentine *M. guttatus* to serpentine populations. Whether such selection promotes accumulation of reproductive barriers through reinforcement or is directly linked with between ecotype-specific reproductive incompatibilities is unknown. However, given experimental evidence for the accumulation of reproductive incompatibilities associated with colonization of serpentines across plant taxa (reviewed by Rajakaruna and Whitton, 2004; Rajakaruna, 2018) the genomics of ecological speciation in serpentine plants promises to be a fruitful area for future research.

## Convergence and Parallelism in Serpentine Adaptation

The patchy, island-like distribution of serpentines provides convenient natural replicates to study repeated evolution both within species (parallelism) and between them (convergence). Given the many independent evolutionary paths that species (and indeed populations within species) have taken to serpentine-adaptive phenotypes, it is reasonable to expect reuse of common pathways, or even proximal molecular actors to the common serpentine challenges.

While phenotypic parallelism and convergence are obvious from a rich literature, only recently has genetic parallelism and convergence been tested. Genetic parallelism has been indicated in *M. guttatus*, where two serpentine populations share a major QTL important for serpentine adaptation (Selby and Willis, 2018). Some indication of genetic parallelism has also been provided by Turner et al. (2010) in European and North American serpentine populations of *A. lyrata*, which shared the same non-synonymous mutation that codes for a change in the *TPC1* locus encoding Ca-ion channel. This is almost fixed in three serpentine populations in two continents. Genetic convergence was described in *A. arenosa* and *A. lyrata* by Arnold et al. (2016) in nine gene coding loci with serpentine-relevant predicted functions, such as Ca, K, and Ni homeostasis (Table 1). Importantly, studies of evolutionary history and fitness responses of several other plant systems, such as *C. alpinum* (Scandinavia – Berglund et al., 2004), *L. californica* complex (California – Rajakaruna et al., 2003a,b,c), and *Solidago virgaurea* (Japan – Sakaguchi et al., 2017, 2019), suggest parallel evolution of serpentine ecotypes, which may be a frequent phenomenon (see also Figure 2). Further leveraging such truly non-model systems for genomic studies should bring novel vital insights into the basis of local adaptation.

**Table 1:** Candidate convergent loci mediating serpentine adaptation in *Alyssum serpyllifolium, Arabidopsis arenosa, Arabidopsis lyrata, and Mimulus guttatus*.

Species	*Arabidopsis thaliana* homolog	Potential function^1^
*Arabidopsis arenosa*^2^ and *Arabidopsis lyrata*^3^	*AT1G51310*	A tRNA-methyltransferase.
	*AT3G10985*	A senescence-associated gene. Expression is induced in response to treatment with Nep1, a fungal protein that causes necrosis. The mRNA is cell-to-cell mobile.
	*AT5G04320*	Encodes a protein that protects meiotic centromere cohesion.
	*AT5G04330*	Cytochrome P450 superfamily protein.
	*AT4G03560*	*TPC1*, a vaculolar Ca^2+^ channel.
	*AT4G19960*	*KUP9*, a K+ ion transmembrane transporter that can also mediate Cs uptake if expressed in *Escherichia coli*.
	*AT5G37710*	Alpha/beta-Hydrolases superfamily protein.
	*AT5G37720*	*ALY4*, a member of a protein family involved in RNA export from the nucleus and transcriptional coactivation.
*A. arenosa*^2^*, A. lyrata*^3^, and *Alyssum serpyllifolium*^4^	*AT5G03570*	*FPN2*, a tonoplast-localized Ni transporter.
*A. lyrata*^3^ and *Mimulus guttatus*^5^	*AT4G19670*	RING/U-box superfamily protein.

1 The *Arabidopsis* Information Resource (TAIR), www.arabidopsis.org, July, 2019.

2 (Arnold et al., 2016).

3 (Turner et al., 2010).

4 (Sobczyk et al., 2017).

5 (Selby, 2014; Selby and Willis, 2018).

An intriguing open question concerns the origin of the variants that repeatedly exhibit signatures of selection in different serpentine populations. Such repeated signatures may arise in three major ways (Stern, 2013; Lee and Coop, 2019): (i) the causal variants might have been repeatedly sampled from the standing variation present in the ancestral population; (ii) they may have been transferred between adapted populations by gene flow; or (iii) they may have arisen by independent *de novo* mutations. That adaptive gene flow may be involved in serpentine adaptation has been indicated in *A. arenosa* and *A. lyrata* despite overall weak genome-wide signal for introgression between these two species (Arnold et al., 2016). Yet, the relative contribution of these pathways to repeated adaptation remains unknown, particularly in closely related populations and thus represents a fruitful area for future research.

## Conclusion

**Figure 3:**
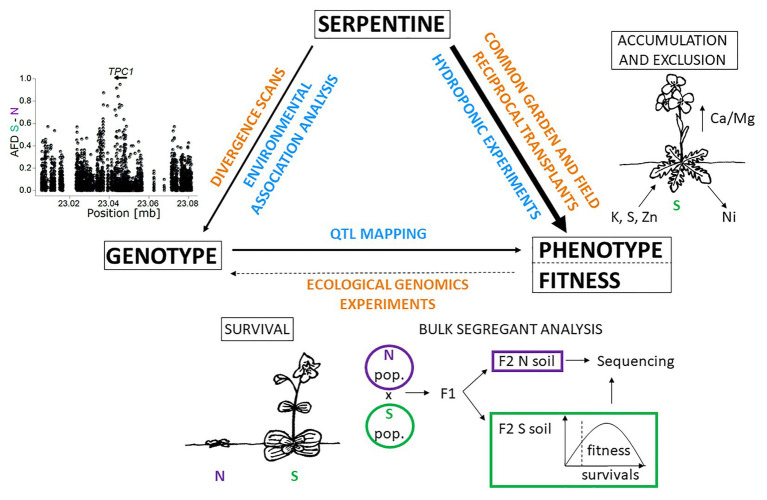
Approaches to study the basis of serpentine adaptation: Integration of holistic (orange) and one factor/hypothesis driven approaches (blue) to investigate links between genotype, phenotype, fitness, and serpentine environments. The right side of the triangle depicts ionomic analysis of *Arabidopsis arenosa* (Arnold et al., 2016), the bottom side is accompanied by the scheme of bulk segregant analysis applied in the recent study of *Mimulus guttatus* (Selby and Willis, 2018), and the left side is supplemented with allele frequency difference graph (AFD) of S and N population for *TPC1* locus Arnold et al. (2016). Line thickness scales with the quantity of empirical studies available. S – serpentine adapted plant, N – non-serpentine plant. The scheme is inspired by Sork et al. (2013) and Rellstab et al. (2015). Illustrations by V. Konečná.

Given the perfect storm of challenges that serpentine presents, a comprehensive understanding of serpentine adaptation is a challenging task that requires multidisciplinary investigation (Wright and Von Wettberg, 2009). However, recent progress in quantitative genetic, ionomic, and population genomic study has shown that understanding the basis for the interacting phenotypes which constitute serpentine adaptation is well worth the effort. Specifically, recent inquiries in the *Arabidopsis* and *Mimulus* genera have provided proof-of-concept that the genetic basis of serpentine adaptation is accessible, and the time is now ripe for synthetic studies (Figure 3). Here, we sketched gaps in our understanding of serpentine adaptation and considered integrative approaches to link candidate adaptive alleles with fitness effects as a promising avenue to make progress.

We suggest that ongoing ecological and population genomic studies of serpentine adaptation hold strong potential to contribute to our understanding of fundamental evolutionary principles, similar to the contributions of early eco-evolutionary studies achieved in the 1950s and 1960s (Kruckeberg, 1950, 1951, 1954, 1967). Aside from the obvious benefit of improved knowledge about soil-stress adaptations for rational crop breeding, serpentine models can efficiently address our understanding of environmentally driven adaptation and ecological speciation. Specifically, serpentine soils represent a strong selective pressure leading to well-tractable adaptive phenotypes. Yet, in contrast to similarly poised mine sites, serpentines provide fully natural setups with longer time frames on which selection has acted. Frequent reports of serpentine endemism and the reproductive isolation experienced by edaphic ecotypes also suggest serpentine plants as attractive, yet still too rarely leveraged models for ecological speciation. Beyond this, serpentine is a highly defined environment characterized primarily by a limited number of soil chemistry components (also with potentially linked biotic interactions). Such “intermediate complexity” makes serpentine an attractive model of adaptation towards a suite of naturally relevant factors, which can still be experimentally manipulated using, e.g., reciprocal transplants. Finally, the repeated origins of serpentine adaptation allow leveraging natural replicates empowering genomic and experimental inference. Genome-wide studies of serpentine populations combined with ecological genomic experiments and/or forward genetic validation may thus bring significant contribution to our general knowledge on the adaptation mechanisms towards environmental challenges.

## Author Contributions

All authors listed have made a substantial, direct and intellectual contribution to the work and approved it for publication.

### Conflict of Interest

The authors declare that the research was conducted in the absence of any commercial or financial relationships that could be construed as a potential conflict of interest.
